# Can left ventricular hypertrophy on electrocardiography detect severe aortic valve stenosis?

**DOI:** 10.1371/journal.pone.0241591

**Published:** 2020-11-04

**Authors:** Takashi Mino, Seishi Kimura, Atsuhiro Kitaura, Tatsushige Iwamoto, Haruyuki Yuasa, Yasutaka Chiba, Shinichi Nakao

**Affiliations:** 1 Department of Anesthesiology, Kindai University Faculty of Medicine, OsakaSayama, Osaka, Japan; 2 Clinical Research Center, Kindai University Hospital, OsakaSayama, Osaka, Japan; International University of Health and Welfare, School of Medicine, JAPAN

## Abstract

**Background:**

Severe aortic stenosis (AS) is increasing in the aging society and is a serious condition for anesthetic management. However, approximately one-third of patients with severe AS are asymptomatic. Echocardiography is the most reliable method to detect AS, but it takes time and is costly.

**Methods:**

Data were obtained retrospectively from patients who underwent surgery and preoperative transthoracic echocardiography (TTE). LVH on ECG was determined by voltage criteria (Sv_1_ + Rv_5_ or _6_ ≥3.5 mV) and/or the strain pattern in V_5_ and V_6_. Severe AS was defined as a mean transaortic pressure gradient ≥40 mmHg or aortic valve area ≤1.0 cm^2^ by TTE.

**Results:**

Data for 470 patients aged 28–94 years old were obtained. One hundred and twenty-six patients had severe AS. LVH on ECG by voltage criteria alone was detected in 182 patients, LVH by strain pattern alone was detected in 80 patients and LVH by both was detected in 55 patients. Multivariable logistic analysis revealed that LVH by the strain pattern or voltage criteria, diabetes mellitus, and age were significantly associated with severe AS. The AUC for the ROC curve for LVH by voltage criteria alone was 0.675 and the cut-off value was 3.84 mm V, and the AUC for the ROC for age was 0.675 and the cut-off value was 74 years old.

**Conclusion:**

Our study suggests that patients who are 74 years old or over with LVH on ECG, especially those with DM, should undergo preoperative TTE in order to detect severe AS.

## Introduction

Severe aortic stenosis (AS) is a significant risk factor for perioperative mortality and morbidity in noncardiac surgery [1–4]. Indeed, according to the 2014 American College of Cardiology/American Heart Association (ACC/AHA) guidelines on the management of valvular heart disease in patients with moderate to severe AS, the 30-day mortality is higher for patients with AS (2.1%) than for propensity score-matched controls (1%) because of the higher risk of post-operative myocardial infarction [5].

The prevalence of AS markedly increases with age, being over 1% in patients 65 years old or over [6]. There are three cardinal symptoms of severe AS: angina, syncope, and dyspnea. However, as symptoms do not correlate well with the severity of stenosis [7] and approximately one-third of patients with severe AS are asymptomatic [8], it may be difficult to detect asymptomatic severe AS preoperatively.

Echocardiography is the most reliable method to detect AS and assess its severity, but it cannot be performed for all patients preoperatively because it takes time and effort, and is costly. An electrocardiogram (ECG) for AS patients often demonstrates a left ventricular hypertrophy (LVH) pattern in response to increased afterload, but traditional ECG criteria for LVH cannot necessarily reliably detect anatomical LVH [9]. However, as ECG is a low-cost and widely available examination, and all patients are required to undergo 12-lead ECG preoperatively in Japan, we investigated whether LVH on ECG can be used as a detector of severe AS in special cohorts.

## Methods

The study was carried out after obtaining institutional approval from the Kindai University Faculty of Medicine Ethics Committee (No. 30–083) and was registered in the University Hospital Medical Information Network (UMIN 000034965), which is accepted by the ICMJE (International Committee of Medical Journal Editors). Researchers who obtained the data were completely different from researchers who analyzed the data.

Data for patients who underwent surgery and preoperative transthoracic echocardiography (TTE) in Kindai University Hospital between January 2013 and December 2017 were used, and demographic, medical history, medication, smoking history, 12-lead ECG, and TTE data were obtained during August 2018 to May 2019 from the electronic medical records. After obtaining the data, all patients’ names were deleted and anonymized for analyzing them. Informed consent forms were obtained from all the patients, and scanned and preserved in the electronic medical records of Kindai University Hospital. LVH on ECG was determined by Sokolow-Lyon voltage criteria (Sv_1_ + Rv_5_ or _6_ ≥3.5 mV) [10] and/or by strain pattern (≥1-mm concave down sloping ST-T segment depression with asymmetrical T-wave inversion) in V_5_ and V_6_ [11, 12] on a standard 12-lead ECG. Severe AS was defined as a mean transaortic pressure gradient (MPG) ≥40 mmHg and/or aortic valve area (AVA) ≤1.0 cm^2^ by TTE according to the 2014 American College of Cardiology/American Heart Association guidelines for the management of patients with valvular heart disease [5].

All statistical analyses were performed using EZR (Saitama Medical Center, Jichi Medical University, Saitama, Japan), which is a graphical user interface for R (The R Foundation for Statistical Computing, Vienna, Austria). More precisely, it is a modified version of R commander designed to add statistical functions frequently used in biostatistics [13]. The sample size was determined by the suggestion that the result of a multivariable logistic regression method should have more than 10 outcome events per independent predictor for accuracy, assuming a power of 0.8, a type 1 error rate of 0.05, a prevalence of 3% [14], and an expected odds ratio of 2.0, and we estimated more than 378 patients to be necessary. To compensate for drop out, data from 494 patients were retrospectively obtained. The exclusion criteria included patients with abnormal ECG, such as, arterial fibrillation, right or left bundle branch block. The normality of continuous valuable data was assessed using the Kolomogorov-Simiov test. Body mass index (BMI) was assessed using the chi-squared test. The multivariable logistic regression analysis was performed to explore factors that affect severe AS [15].

For the factors selected as those affecting severe AS by the logistic regression analysis, a receiver operating characteristic curve (ROC) was generated, and the area under curve (AUC) was calculated to assess the extent of affection to severe AS. The optimal cut-off point was determined as the point on the curve closest to the upper left corner of the graph. By the optimal cut-off point, the continuous variable was converted to a binary variable; then, univariable logistic regression analysis was performed with the binary variable as an independent variable.

## Results

**Fig 1:**
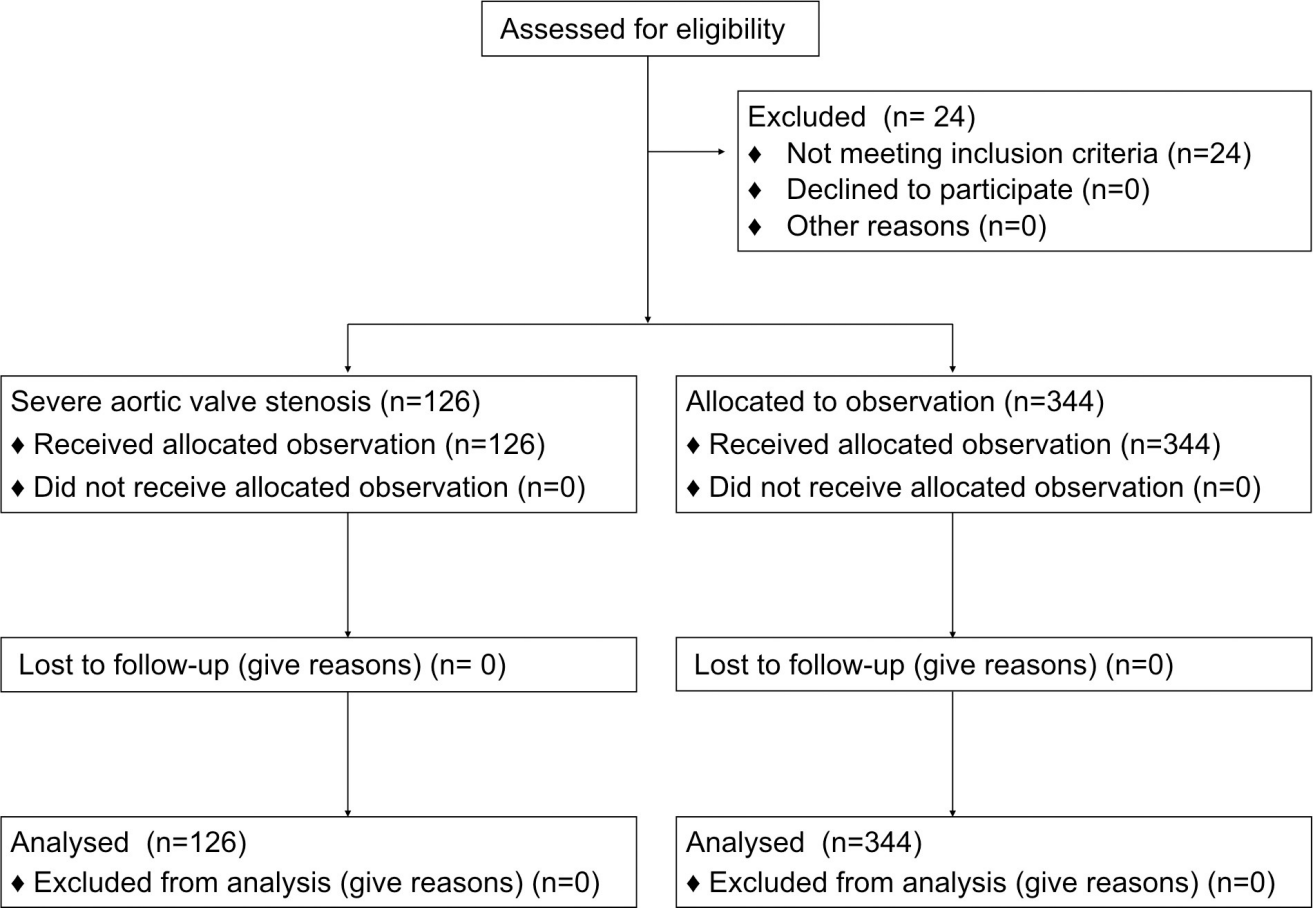
The structured patient flow chart for the experiment.

Data from 470 patients out of 494 patients aged 28–94 years old who underwent preoperative TTE (205 for cardiac surgeries, 186 for orthopedic surgeries, 45 for abdominal or thoracic surgeries, and 34 other surgeries) were actually used (Fig 1).

The baseline characteristics of the study population are shown in Table 1. Forty-five patients were <60 years old, 290 patients were between 60 years old or over and under 80 years old, and 135 patients were ≥80 years old (median: 75 years old).

**Table 1:** Baseline characteristics of the patients.

	Total	<60 y	60–79 y	≥80 y
Number of patients	470	45	290	135
Male/female	200/270	24/21	127/163	49/86
Height (cm, mean ± SD)	155 ± 11	162 ± 9	155 ± 9	151 ± 9
Weight (kg, mean ± SD)	56 ± 12	63 ± 16	56 ± 12	52 ± 11
Diabetes mellitus	64	7	42	15
Hemodialysis	10	0	6	4
Hypertension	129	4	81	43
Smoking	44	1	24	19
Type of surgery				
Cardiac surgery	205	23	167	15
Orthopedic surgery	186	16	165	5
Abdominal or thoracic surgery	45	3	41	1
Neurosurgery	10	0	9	1
Otorhinolaryngologic surgery	8	0	8	0
Plastic surgery	7	1	6	1
Gynecological surgery	5	1	4	0
Urological surgery	4	0	4	0

Of all 470 patients, 126 had severe AS. Table 2 shows the number of the severe AS patients in each surgical category. Naturally, cardiac surgical patients accounted for the majority of the severe AS patients.

**Table 2:** The number of patients with severe aortic stenosis in each surgical category.

Type of surgery	Number of patients
Cardiac surgery	114
Orthopedic surgery	3
Abdominal or thoracic surgery	7
Neurosurgery	1
Otorhinolaryngologic surgery	1
Plastic surgery	0
Gynecological surgery	0
Urological surgery	0
Total	126

Of all 470 patients, LVH on ECG was detected by the Sokolow-Lyon voltage criteria alone in 182, by the strain pattern alone in 80 patients, and by both in 55 patients. Of 45 patients <60 years old, it was detected by the Sokolow-Lyon voltage criteria alone in 14 patients, by the strain pattern alone in 9 patients, and by both in 5 patients. Of 290 in patients between 60 years old or over and under 80 years old, it was detected by the Sokolow-Lyon voltage criteria alone in 107 patients, by the strain pattern alone in 46 patients, and by both in 31 patients. Of 135 patients ≥80 years old, it was detected by the Sokolow-Lyon voltage criteria alone in 61 patients, by the strain pattern alone in 25 patients, and by both in 19 patients (Table 3).

**Table 3:** The number of patients with severe Aortic Stenosis (AS) and Left Ventricular Hypertrophy (LVH) on Electrocardiogram (ECG).

	Total	<60 y	60–79 y	≥80 y
Number of patients	470	45	290	135
Severe AS	126	3	66	57
LVH on ECG by voltage criteria alone	182	14	107	61
LVH on ECG by strain pattern alone	80	9	46	25
LVH on ECG by voltage criteria + strain pattern	55	5	31	19

The sensitivities, specificities, positive predictive values, and negative predictive values of the Sokolow-Lyon voltage criteria alone, the strain pattern alone, and both for severe AS in all ages, in those <60 years old, in those ≥60 years old, and in those ≥80 years old are shown in Table 4.

**Table 4:** Sensitivity, specificity, positive predictive value, and negative predictive value of left ventricular hypertrophy on electrocardiography for severe aortic valve stenosis in each age bracket.

	Sensitivity	Specificity	Prevalence	Positive Predictive Value	Negative Predictive Value
All ages					
Voltage criteria +strain pattern	66.67%	64.24%	26.81%	39.27%	83.60%
Voltage criteria alone	60.32%	69.19%	26.81%	41.76%	82.63%
Strain pattern alone	33.33%	88.95%	26.81%	52.49%	78.45%
< 60 years old					
V voltage criteria + strain pattern	33.33%	59.52%	6.67%	25.11%	68.67%
Voltage criteria alone	33.33%	69.05%	6.67%	30.48%	71.77%
Strain pattern alone	33.33%	80.95%	6.67%	41.60%	74.88%
≥ 60 years old					
Voltage criteria + strain pattern	67.48%	64.90%	28.94%	43.91%	83.05%
Voltage criteria alone	39.02%	60.26%	28.94%	28.56%	70.81%
Strain pattern alone	33.33%	90.07%	28.94%	57.75%	76.83%
≥ 80 years old					
Voltage criteria + strain pattern	66.67%	62.82%	42.22%	56.71%	72.06%
Voltage criteria alone	61.84%	66.67%	42.22%	57.55%	70.51%
Strain pattern alone	26.32%	87.18%	42.22%	60.01%	61.82%

Multivariable logistic analysis revealed the strain pattern (odds ratio (OR) = 3.1; 95% confidence interval (CI) = 1.71–5.60; p = 0.00018), the Sokolow-Lyon voltage criteria (OR = 1.43; 95% CI = 1.18–1.73; p = 0.00025), diabetes mellitus (DM) (OR = 2.82; 95% CI = 1.41–5.63; p = 0.0033), and age (OR = 1.08; 95% CI = 1.05–1.11; p = 0.00000029) to be significantly associated with severe AS (Table 5).

**Table 5:** Odds ratio and 95% confidence interval of each variable.

Variables	Estimate	Odds Ratio	95% Confidence Interval	P Value
(Intercept)	-5.80375	0.00 (0.00–0.05)		0.000072
Diabetes mellitus	1.0365	2.82	1.41–5.63	0.0033
Hypertension	-0.08014	0.92	0.54–1.59	0.77
LVH on ECG by Sokolow-Lyon voltage criteria	0.35646	1.43	1.18–1.73	0.00025
LVH on ECG by strain pattern	1.13122	3.1	1.71–5.60	0.00018
Smoking	-0.1633	0.85	0.43–1.67	0.64
Gender	-0.19784	0.82	0.47–1.42	0.48
Hemodialysis	-0.69396	0.5	0.10–2.41	0.39
Age	0.07317	1.08	1.05–1.11	0.00000029

LVH: Left Ventricular Hypertrophy; ECG: Electrocardiogram.

**Fig 2:**
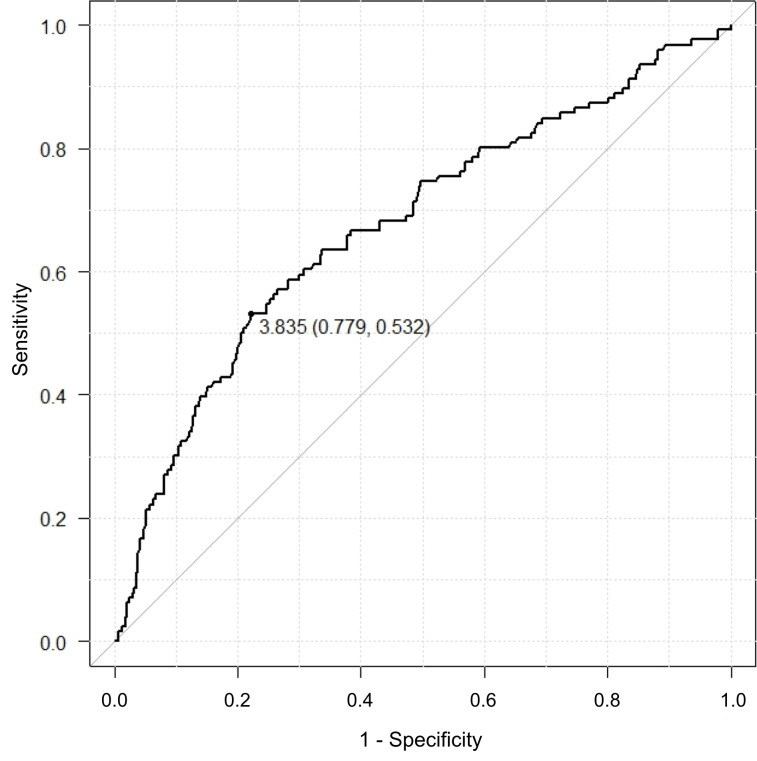
Receiver operating characteristic curve (ROC) for left ventricular hypertrophy on electrocardiogram by the Sokolow-Lyon voltage criteria. The cut-off value is 3.835, specificity is 0.779, and sensitivity is 0.532.

**Fig 3:**
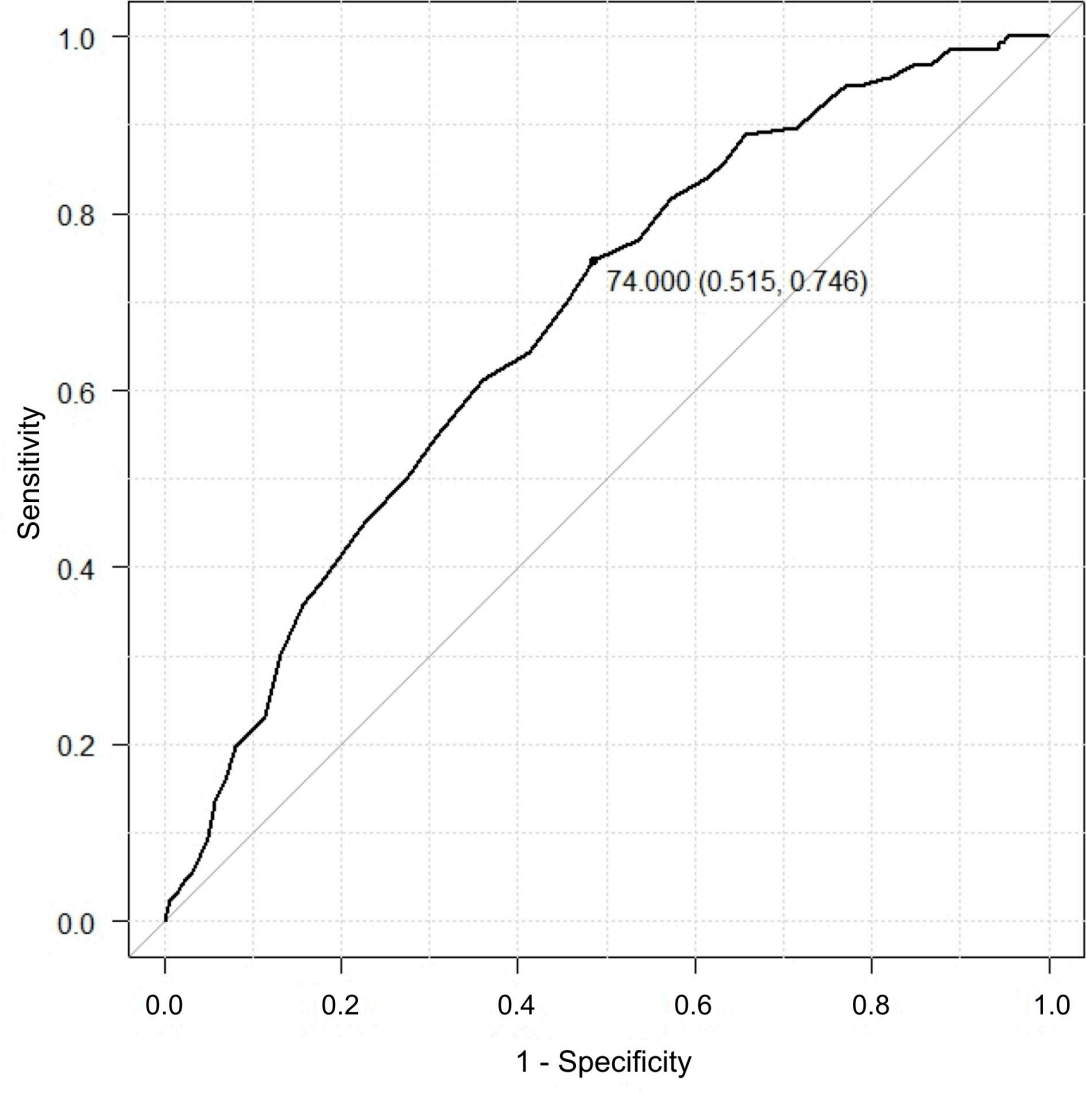
Receiver operating characteristic curve (ROC) for age. The cut-off value is 74.0, specificity is 0.515, and sensitivity is 0.746.

On the basis of ROC curves, the AUC for LVH on ECG by the Sokolow-Lyon voltage criteria alone was 0.675 (95% CI = 0.622–0.728), where the optimal cut-off value was 3.84 mm V (Fig 2). The AUC for age was 0.675 (95% CI = 0.618 − 0.733), where the optimal cut-off value was 74 years old (Fig 3).

Table 6 shows the results of univariable logistic regression analysis with a binary variable categorized by those cut-off values as the independent variable.

**Table 6:** Odds ratio and 95% confidence interval of each variable based on the cut-off values.

Variables	Estimate	Odds Ratio	95% Confidence Interval	P Value
(Intercept)	-0.3488	0.706	0.142	0.67
Diabetes mellitus	1.0928	2.98	1.48	0.00221
Hypertension	-0.0121	0.988	0.57	0.966
LVH on ECG by Sokolow-Lyon voltage criteria	1.18326	3.26	1.99	0.00000251
LVH on ECG by strain pattern	1.18842	3.28	1.84	0.0000603
Smoking	-0.38346	0.681	0.346	0.267
Gender	-0.18388	0.832	0.482	0.509
Hemodialysis	-0.70336	0.495	0.0996	0.39
Age	1.23692	3.44	2.06	0.00000223

LVH: Left Ventricular Hypertrophy; ECG: Electrocardiogram.

## Discussion

In the present study, we demonstrated that LVH on ECG by both the Sokolow-Lyon voltage criteria and the strain pattern, age, and DM is significantly associated with severe AS. There was a poor association between age and severe AS and between LVH on the ECG by the Sokolow-Lyon voltage criteria alone and severe AS on ROC curves, because the AUCs for the ROC curve for age was 0.675 and for the LVH was 0.675. However, those values would increase if the number of patients is increased. Our study suggests that elderly patients (74 years old or over) with LVH on ECG by the Sokolow-Lyon voltage criteria and/or the strain pattern, especially those with DM, should receive preoperative TTE in order to detect severe AS.

Although perioperative mortality rates in patients with AS undergoing noncardiac surgery have decreased markedly with advancements in anesthesia and intensified medical therapy [1, 2], and several reports demonstrated no significant differences in both the perioperative mortality rate and major adverse cardiovascular events between patients with asymptomatic severe AS and control patients without AS undergoing noncardiac surgery [16–18], AS in those patients, even asymptomatic AS, was diagnosed before surgery and appropriate perioperative management must have been performed. It is imperative for anesthesiologists to know preoperatively whether a patient has severe AS in order to perform safe anesthetic management during and after surgery. However, as approximately one-third of patients with severe AS are asymptomatic [8], we need to detect patients with severe AS preoperatively using routine medical examinations. As preoperative 12-lead ECG examination is required for adults in Japan, it is reasonable for anesthesiologists to try to detect severe AS using the ECG. Ehara et al. reported that LVH on ECG by the Sokolow-Lyon voltage criteria has little association with anatomical LVH, whereas the strain pattern on ECG is significantly associated with the presence of anatomical LVH [19]. In contrast, Greve et al. reported that LVH on ECG by the Sokolow-Lyon voltage criteria and the strain pattern were independently associated with the peak aortic jet velocity and LV mass index [20], and were independently predictive of a poor prognosis in patients with asymptomatic AS [21]. Furthermore, Shah et al. demonstrated that the strain pattern on ECG is a specific marker of midwall myocardial fibrosis and predicts adverse clinical outcomes in AS [12]. Our results confirm these reports that LVH on ECG by the Sokolow-Lyon voltage criteria and strain pattern is associated with AS and its pathological conditions. Auscultation is a convenient and useful test for the detection of AS, but unfortunately we do not have auscultatory findings data.

The prevalence of AS is well known to markedly increase with advanced age [6], and Osnabrugge et al. reported that the prevalence of all AS in elderly patients (>75 years) was 12.4% (95% CI: 6.6% to 18.2%) and that of severe AS was 3.4% (95% CI: 1.1% to 5.7%) [14]. In the present study, we confirmed that age was significantly associated with severe AS and the cut-off value was 74 years old. Based on previous reports and our current results, we recommend that patients aged 74 years old or over with LVH by the Sokolow-Lyon voltage criteria and strain pattern on ECG, especially those with DM, undergo preoperative TTE even if they have no cardiac symptoms.

Several potential limitations of this study should be considered: First, as this study was a retrospective observational study and only patients who underwent preoperative TTE were enrolled, most patients had cardiac diseases and/or were elderly. Second, only patients with severe AS, not mild to moderate AS, were investigated.

## Conclusions

We confirmed that LVH on ECG by the Sokolow-Lyon voltage criteria or the strain pattern was independently associated with severe AS, and that DM and age are also associated with severe AS. We strongly recommend that elderly patients who are ≥74 years old with LVH on ECG by the Sokolow-Lyon voltage criteria or strain pattern, especially those with DM, undergo preoperative TTE in order to detect severe AS, even if they have no symptoms.

## Supporting information

S1 Checklist TREND checklist. (PDF)

S1 Protocol Research protocol (Japanese). (DOCX)

S2 Protocol Research protocol (English). (DOCX)

S1 Data All data underlying the findings described in the manuscript. (XLSX)

S1 File Certificate approval of our institution (Kindai University Hospital). (PDF)
