# Supplementary material for: Can left ventricular hypertrophy on electrocardiography detect severe aortic valve stenosis?
Source: PLoS One. 2020 Nov 4;15(11):e0241591. doi: 10.1371/journal.pone.0241591 (PMC7641401; doi:10.1371/journal.pone.0241591)
Supplement: S1 Protocol — (DOCX) [file pone.0241591.s002.docx]

研究計画書

課題名：

術前心電図で左室肥大を示す患者における大動脈弁狭窄症合併率の検討（後向き研究）

1.0版　2018年5月24日

１．背景

現在、日本国内における手術前における心臓のスクリーニング検査は、一般的に安静時１２誘導心電図である。心臓超音波検査などの検査は非常に手間とコストがかかるため、心電図で異常を認めた場合のみ行うことが多い。心電図における左室肥大所見は、日常的にみられる所見で、高血圧患者などの一般的な患者で認め、あまり注意されないこともある一方で、同様に心筋肥大を伴う大動脈弁狭窄症や肥大型心筋症といった麻酔管理に大きく制限を加えうる合併症においてもみられる所見である。大動脈弁狭窄症や肥大型心筋症などの術前検査における未検出はしばしば認められるが、これらは重大な周術期事故の原因となりかねない。また、内科診療においても、心電図所見と大動脈弁狭窄症や肥大型心筋症といった疾患の疫学的関係が明らかになれば、診断や早期発見の一助となりうる上に、心臓超音波検査の適正使用により医療費の削減につながる可能性もあると考えられる。

２．目　的

大動脈弁狭窄症は麻酔管理上特に注意を要する合併症の１つである。術前心電図における左室肥大はしばしば認められる所見であるが、時に大動脈弁狭窄症などの危険な合併症の存在を示唆している可能性がある。今回の研究においては、術前心電図で左室肥大を認める患者における大動脈弁狭窄症の割合を検討するに加え、どのような因子が強く大動脈弁狭窄症の存在と相関するかについても検討する。

３．研究の評価項目

対象の12誘導心電図、心臓超音波検査の結果、及び年齢、性別、身長、体重、body-mass-index (BMI)、既往歴、内服薬を評価項目とする。心電図における左室肥大所見及び大動脈弁狭窄症についての疫学的検討及び、その他の危険因子について評価する。

４．研究計画・研究デザイン

　デザイン名：後向き症例対象研究

５．対象

当院にて麻酔科管理で手術を受け、所定の術前検査を施行されている患者。

本人もしくは代理人によって本研究への参加拒否の意思が示された場合は除外する。

６．研究のアウトライン

　対象症例群から、参加拒否の意思表示があった症例を除外したのち、術前に施行された12誘導心電図において、左室肥大（voltage criteriaとV5からV6のstrainパターン）を呈するかどうかで左室肥大群と非左室肥大群の2群に分ける。そのうえで、心臓超音波検査の結果を基に、大動脈弁狭窄症の有無を確認し、群内の有病率について検討する。また、年齢、性別、身長、体重、body-mass-index (BMI)、既往歴、内服薬を検討することで、その他の関連因子についても検討する。それぞれの因子については多変量解析法を用いて評価する。

７．研究の中止基準

以下のような場合には研究を中止する．研究を中止した場合には，その理由を明らかにする．

1. 担当医師が研究の継続が困難と判断した場合
2. 患者から研究中止の申し出があった場合

８．登録方法

研究分担者により適宜患者リストに登録する．

９．実施期間

倫理委員会承認日より向う５年間

対象は2013年1月から2017年12月までの症例とする。

１０．目標症例数

一般に日本における大動脈弁狭窄症の罹患率は3％前後とされている。これを心電図及び心エコーにより診断する場合の精度評価を行う。推定Area Under the Curve (AUC)を0.8、検出力0.8、有意水準5％とし仮定した場合、ROC解析に必要な症例数は236例となる。そのため、目標症例数は236例とする。当院では大動脈弁狭窄症に対する大動脈弁置換術が平均40件/年、大動脈弁狭窄症合併患者の非心臓手術が平均60件/年ある。また、左室肥大所見を呈する患者は0.5～1件/日程度存在することから、無理なく目標症例数を達成可能である。

１１．解析方法

集積できたデータに基づき，各評価項目について多変量解析を用いて相関関係を検討するものとする．

１２．記録の保存

　12-1　保存すべき資料

　　　　研究に参加した患者の患者情報と対応表を保存する．

　12-2　保存期間及び保存場所，保存責任者

　　　　保存期間：研究終了後5年間，　保存場所：麻酔科医局

　　　　保存責任者：法里　慧　　所属：麻酔科学講座 職名：医学部助教

　12-3　患者のプライバシー保護

　症例報告書等の作成，取扱いにおいては，患者のプライバシー保護に配慮する．

　　　　患者は患者別コードを用い個人の特定をできないようにする．

１３．健康被害に対する補償

通常の診療範囲内で行われた検査結果を用いた研究であり、本研究によって健康被害が生じる可能性はない。

１４．予測される医療費

本臨床研究に参加することで新たに生じる医療費はないものと考えられる．

１５．患者に対する金銭の支払い等

該当しない

１６．研究資金及び利益相反

研究資金としては麻酔科講座費を使用する．外部資金は導入しない．利益相反はない．

１7．倫理的事項

遵守すべき諸規則として，「世界医師会ヘルシンキ宣言」「人を対象とする医学系研究に関する倫理指針（平成29年2月28日一部改正）」の最新のものに従う．

１８．健康被害に対する補償について

　　本研究の実施に起因して有害事象が発生する可能性はない．

１９．プロトコルの承認

研究計画書は，近畿大学医学部倫理委員会の審査を受け，承認されたのちに発効する．その後近畿大学医学部長の許可を得て研究を実施する．

２０．プロトコルの改訂

主任研究者は研究開始後にプロトコル改訂が必要となった場合には，プロトコルの改訂を行う．改訂を行う場合は近畿大学医学部倫理委員会にて審査を受けなければならない．改訂後，主任研究者は改訂後の関連資料を研究分担者に送付する．

２１．研究の中止及び終了

　研究の終了

　　追跡期間の終了及びすべての症例情報の収集と解析の終了をもって研究の終了とする．主任研究者は研究終了を研究分担者に報告し，近畿大学医学部長をはじめとする関連部門に報告する．

　研究の早期中止

　　近畿大学医学部倫理委員会からの提言もしくはその他の理由により，研究の早期中止を決定した場合には，その理由及び以後の対応についてただちに研究分担者に連絡する．加えて，医学部長及び関連部門に当該研究の早期中止及びその理由を報告する．

２２．組織

　　臨床研究実施機関

　　近畿大学医学部　麻酔科学講座

　主任研究者（臨床研究の統括）

　　近畿大学医学部　麻酔科学講座　主任教授　中尾　慎一

　　大阪府大阪狭山市大野東377-2

<TEL:072-366-0221（代表>）　内線：3238　FAX:072-365-1662

　研究分担者

氏名：北浦　淳寛　所属：麻酔科学講座 職名：医学部助教

氏名：木村　誠志　所属：麻酔科学講座 職名：医学部助教

氏名：美野　多佳志　所属：麻酔・疼痛制御・集中治療学　職名：大学院生

氏名：濱崎　真一　所属：麻酔科学講座 職名：医学部助教

２３．結果の発表及び出版

　本臨床研究の結果は，主任研究者と研究分担者が協議して著者を選出する．本臨床試験で得られた見地は国内外の麻酔科・集中治療領域の学会で発表及び論文投稿を行う．

付記：研究対象者からの相談への対応について

　　研究対象者から相談があった場合は主任研究者及び研究分担者が対応する。相談手段を確保するため、倫理委員会承認後、当科ホームページに掲載するオプトアウトのページに連絡先を明示する。なお、研究に関する資料の閲覧は研究の独創性を損なわない範囲で個別に検討する。
