# Supplementary material for: Can left ventricular hypertrophy on electrocardiography detect severe aortic valve stenosis?
Source: PLoS One. 2020 Nov 4;15(11):e0241591. doi: 10.1371/journal.pone.0241591 (PMC7641401; doi:10.1371/journal.pone.0241591)
Supplement: S2 Protocol — (DOCX) [file pone.0241591.s003.docx]

Study protocol

Title of the research:

Can left ventricular hypertrophy on the electrocardiography detect severe aortic valve stenosis? (retrospective study)

May 24, 2018

１．Background:

The 12-lead electrocardiogram (ECG) is a must for preoperative screening examination for adults in Japan. However, cardiac echocardiography is not routinely performed preoperatively, because it takes time and is costly. Severe aortic valve stenosis (AS) is increasing in the aging society and is a serious condition for anesthetic management, but one third of it is asymptomatic and the severity of AS is not necessarily correlated with the severity of symptoms. Left ventricular hypertrophy (LVH) on the ECG represents excessive afterload to the left cardiac ventricle, and is sometimes caused by sever AS. There have been already some studies which investigate whether LVH on the ECG can detect severe AS or not, and the results are controversial. However, few reports have investigated what patients with the LVH on the ECG most likely have severe AS and what kinds of risk factors are involved in the close relation between the LVH and the severe AS. We are sure that this study can contribute to the safety of patients who undergo surgery and the reduction of medical expenses.

２．Objectives:

To investigate whether the LVH on the ECG can detect severe AS and what kinds of risk factors are involved in the anatomic severe AS.

３．Evaluation items:

Following items are evaluated: 12-lead electrocardiogram, echocardiogram, age, sex, height, the weight, body-mass-index (BMI), past history, medications. Data are obtained from an electric medical chart. Epidemiological studies about the LVH on the ECG, sever AS detected with echocardiography, and their risk factors.

４．Research plan and research design:

Design name: retrospective case-control study

５．Objects:

Patients who underwent an operation and had required examinations, and gave us a written informed consent to use their data.

６．Outline of the research:

The LVH is determined by voltage criteria (Sv_1_ + Rv_5_ or _6_ ≥3.5 mV) and/or the strain pattern in V_5_ and V_6_. The sensitivities, specificities, positive predictive values, and negative predictive values of the Sokolow-Lyon voltage criteria alone, the strain pattern alone, and both for severe AS are measured. Severe AS is defined as a mean transaortic pressure gradient (MPG) ≥40 mmHg and/or aortic valve area (AVA) ≤ 1.0 cm^2^ by transthoracic echocardiograph (TTE). Logistic regression models are used to identify significant predictors of severe AS in addition to LVH, such as age, gender, height, body weight, BMI, complications, and medications. In order to estimate the goodness-of-fit of the model, a receiver operating characteristic curve (ROC) is generated, and the area under curve (AUC) is calculated. The cut-off point with the best discrimination is the point on the curve closest to the upper left corner of the graph.

７．Cancellation criteria of the research:

The research is cancelled in the following cases and the reasons of the cancellation should be clarified.

1) When a researcher concerned judges continuation of the study to be difficult.

2) When a patient asks the cancellation of the study.

８．Registration method:

Participants of the research are enrolled in the list by a researcher appropriately.

９．Research period:

From January, 2013 to December, 2017.

10. The goal number of cases:

Generally, the prevalence of the aortic stenosis in Japan is around 3%. When estimated area under the curve (AUC) is set to be 0.8, detection power 0.8, a level of significance 5%, the number of cases necessary for ROC analysis becomes 236 cases. Therefore, the goal number of cases is estimated to be 236 cases. In our hospital, as there are at least 40 cases/year of aortic valve replacement operations for AS and at least 60 cases/year of non-cardiac operations in patients with AS, and there are around 0.5-1 case/day of LVH on the ECG, the goal number seems reasonable and the study can be done during the set period.

11. Method of analysis:

In order to investigate a correlation between LVH on the ECG and severe AS, and risk factors for severe AS, a multivariate analysis is used.

12. Record preservation:

12-1. The document which should be stored:

The patient information and collected data

12-2. Preservation period and preservation place, manager for preservation:

Preservation period: for five years after the study ends.

Preservation place: The dispensary of the department of anesthesiology

The manager for preservation: Kei Houri (an assistant professor of anesthesiology)

12-3 Privacy protection of the patient:

The privacy of the participant patients should be strictly protected.

An individual cord number for a patient is used in order to prevent personal identification.

13. Compensation for the health hazard:

Participation in this research presents no possibility of health hazards, because the research uses results of patient’s physical examination carried out in an ordinary clinical setting.

14. The medical expenses that are predicted:

No extra medical expenses by participating in this research.

15. Payment of the money for the participant patients:

No.

16. A study fund and conflict of interest:

A fund of the department of anesthesiology is used, and an external fund is not used. There is no conflict of interest.

17. Ethics

We will strictly follow the latest version of the " Helsinki Declaration by World Medical Association " and " Ethical Guidelines for Medical and Health Research Involving Human Subjects (February 28, 2017 some revisions).

18. About the compensation for the health hazard:

There is no possibility of health hazards to the participant patients.

19. Approval of the protocol:

After receiving the inspection and obtaining the approval from the Kindai University Faculty of Medicine Human Subject Review Committee, the study plan takes effect. Researchers will start the study after obtaining the permission of dean of the Kindai university Faculty of Medicine.

20. Revision of the protocol:

When protocol revision is necessary after the study start, the chief researcher revises the protocol. The revision must receive the inspection of the Kindai University Faculty of Medicine Human Subject Review Committee. After obtaining approval, the chief researcher sends related documents to the co-researchers.

21. The cancellation or the end of the research:

21-1. The end of the research:

The end of the research is the end of the research period or the end of the collection and the analysis of all information. The chief researcher reports the end of the research to co-researches and relevant sections including dean of the Kindai University Faculty of Medicine.

21-2. The early cancellation of the research:

When the chief researcher decides the early cancellation of the study due to a proposal from the Kindai University Faculty of Medicine Human Subject Review Committee or other reasons, the chief researcher informs co-researchers of the reason and following responses promptly. In addition, the chief researcher informs dean of the Kindai University Faculty of Medicine and relevant sections of the early cancellation.

22. Organization:

- Institution where the study is performed:

Department of Anesthesiology, Kindai University Faculty of Medicine,

- Chief researcher (supervise the research)

Shinichi Nakao, MD, PhD (Professor and Chair)

Department of Anesthesiology, Kindai University Faculty of Medicine,

377-2, OhnoHigashi, Osaka-Sayama, Osaka

TEL: 072-366-0221, extension: 3238, FAX: 072-365-1662

- Researchers:

Atsuhiro Kitaura, MD: assistant professor, department of anesthesiology

Seishi Kimura, MD, PhD: assistant professor, department of anesthesiology Takashi Mino, : graduate student, department of anesthesiology

Shinichi Hamasaki, MD, PhD: assistant professor, department of

anesthesiology

23. The announcement and the publication of the results:

The chief researcher and co-researchers discuss the results of this research and elect a first author, and the results are presented at a domestic or a foreign meeting of anesthesiology or intensive care medicine, and published in a domestic or a foreign journal.

An additional note: Response to consult from a study participant:

When there is a consultation from a participant, the chief researcher or a researcher responds to it. After the approval of Kindai University Faculty of Medicine Human Subject Review Committee, contact information is clearly stated on the opt-out page which is carried in the homepage of department of anesthesiology. In addition, a permission for reading of the document about the research will be investigated individually as far as the originality of the study is not spoiled.
