# Supplementary material for: Can left ventricular hypertrophy on electrocardiography detect severe aortic valve stenosis?
Source: PLoS One. 2020 Nov 4;15(11):e0241591. doi: 10.1371/journal.pone.0241591 (PMC7641401; doi:10.1371/journal.pone.0241591)
Supplement: S1 File — (PDF) [file pone.0241591.s005.pdf]

Certificate of Approval

Ethics Committee

Kindai University Faculty of Medicine

The following protocol was approved at Aug.8,2018

Title of Protocol
Can left ventricular hypertrophy on electrocardiography detect severe aortic valve stenosis?

Protocol Identification Number
30-083

Principal Investigator
Shinichi Nakao Professor and Chair
Department of Anesthesiology, Kindai University Faculty of Medicine

Name : Itaru Matsumura
Title : Dean, Kindai University Faculty of Medicine

[illegible] May 25, 2020
Signature Date

Name : Osamu Shirakawa
Title : Chair
Ethics Committee of Kindai University Faculty of Medicine

[illegible] May 22, 2020
Signature Date

Certificate of Approval

Ethics Committee

Kindai University Faculty of Medicine

The following protocol was approved at Aug.8,2018

Title of Protocol
Can left ventricular hypertrophy on electrocardiography detect severe aortic valve stenosis?

Protocol Identification Number
30-084

Principal Investigator
Shinichi Nakao Professor and Chair
Department of Anesthesiology, Kindai University Faculty of Medicine

Name : Itaru Matsumura
Title : Dean, Kindai University Faculty of Medicine

Signature

May [illegible], 2020
Date

Name : Osamu Shirakawa
Title : Chair
Ethics Committee of Kindai University Faculty of Medicine

Signature

May 22, 2020
Date
